# High chloride induces aldosterone resistance in the distal nephron

**DOI:** 10.1111/apha.14246

**Published:** 2024-10-24

**Authors:** Helga Vitzthum, Nina Hauswald, Helena Pham, Leya Eckermann‐Reimer, Catherine Meyer‐Schwesinger, Heimo Ehmke

**Affiliations:** ^1^ Department of Cellular and Integrative Physiology University Medical Center Hamburg‐Eppendorf Hamburg Germany; ^2^ DZHK (German Center for Cardiovascular Research) Partner site Hamburg/Kiel/Lübeck Hamburg Germany

**Keywords:** aldosterone, anion, ENaC, mineralocorticoid receptor, potassium diet

## Abstract

**Aim:**

Increasing the dietary intake of K^+^ in the setting of a high salt intake promotes renal Na^+^ excretion even though K^+^ concurrently enhances the secretion of aldosterone, the most effective stimulus for renal Na^+^ reabsorption. Here, we questioned whether in the high salt state a mechanism exists, which attenuates the aldosterone response to prevent renal Na^+^ reabsorption after high K^+^ intake.

**Methods:**

Mice were fed diets containing varying amounts of Na^+^ combined with KCl or KCitrate. Murine cortical connecting duct (mCCDcl1) cells were cultured in media containing normal or high [Cl^−^]. The response to aldosterone was analyzed by high‐resolution imaging and by biochemical approaches.

**Results:**

The canonical cellular response to aldosterone, encompassing translocation of the mineralocorticoid receptor (MR) and activation of the epithelial Na^+^ channel ENaC was repressed in Na^+^‐replete mice fed a high KCl diet, even though plasma aldosterone concentrations were increased. The response to aldosterone was restored in Na^+^‐replete mice when the extracellular [Cl^−^] increase was prevented by feeding a high KCitrate diet. In mCCDcl1 cells, an elevated extracellular [Cl^−^] was sufficient to disrupt the aldosterone‐induced MR translocation.

**Conclusion:**

These findings indicate a pivotal role for extracellular [Cl^−^] in modulating renal aldosterone signaling to adapt MR activation by a high K^+^ intake to the NaCl balance. An impairment of [Cl^−^]‐mediated aldosterone resistance may contribute to excessive MR activation by aldosterone in the presence of a high salt intake characteristic of the Western diet, resulting in an inappropriate salt reabsorption and its downstream detrimental effects.

## 1 INTRODUCTION

**FIGURE 1:**
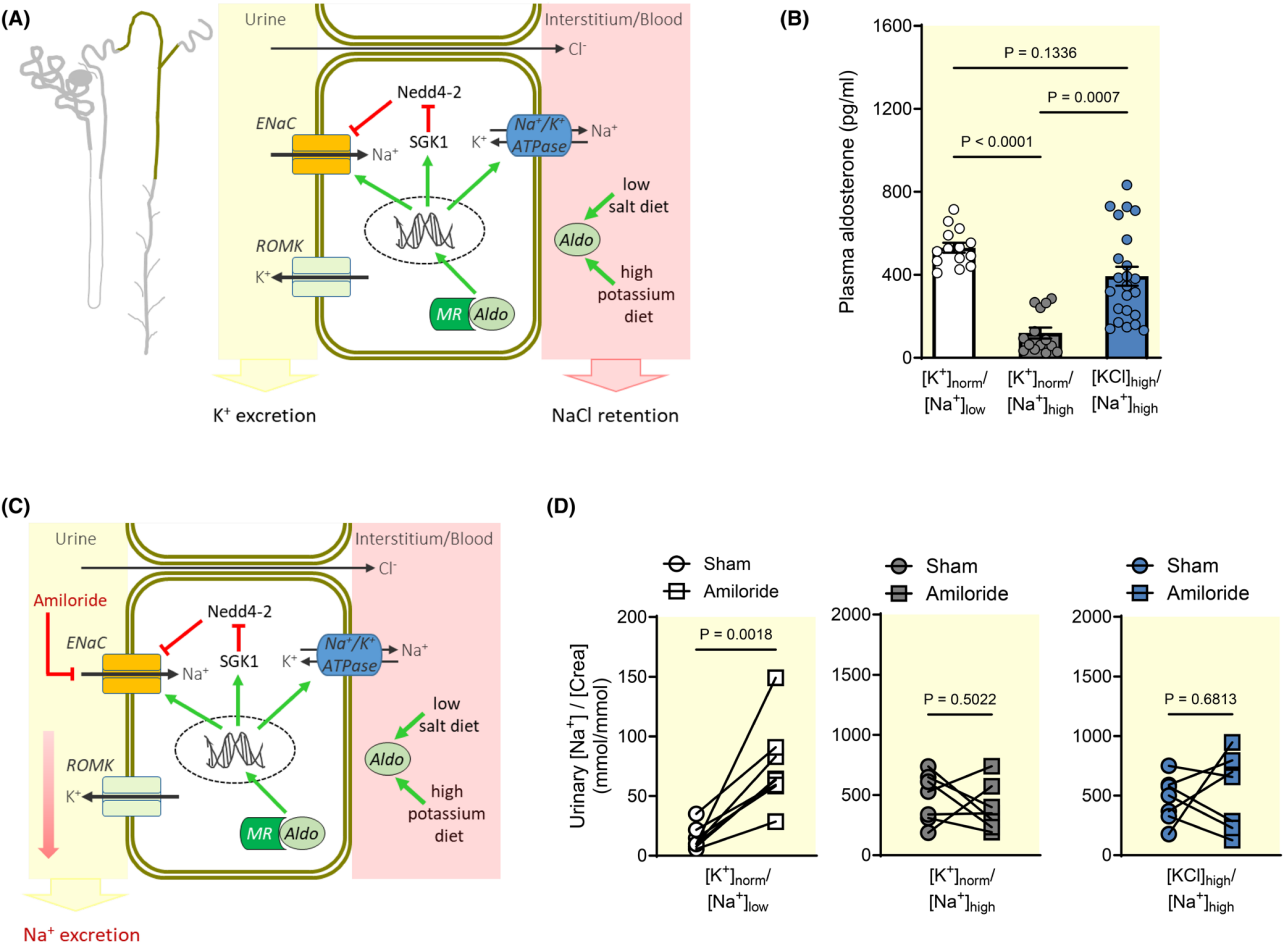
Amiloride fails to induce natriuresis in Na^+^‐replete mice treated with a high KCl diet (A) Canonical aldosterone‐MR‐ENaC pathway induced by low salt (Na^+^) or high potassium (K^+^) diets in the aldosterone‐sensitive distal nephron (ASDN). MR: Mineralocorticoid receptor, ENaC: Na^+^ channel, ROMK: K^+^ channel, SGK1: Serum/glucocorticoid‐regulated kinase 1, Nedd4‐2: E3‐ubiquitin protein ligase. (B) In the Na^+^‐depleted group ([K^+^]_norm_/[Na^+^]_low_) as well as in the high KCl group ([KCl]_high_/[Na^+^]_high_), plasma aldosterone concentrations were significantly increased (mean ± SEM, *n* = 14–23 each) as compared to the normal K^+^ group ([K^+^]_norm_/[Na^+^]_high_). After 10 days of diet, plasma aldosterone levels were not significantly different between the Na^+^‐depleted and the high KCl group (Kruskal–Wallis test followed by Dunn's multiple comparisons test). (C) Schematic illustration of the effect of ENaC inhibition by amiloride on urinary Na^+^ excretion. (D) Treatment with the ENaC blocker amiloride (1.4 mg/kg s.c. in 0.25 mL 0.9% NaCl) increased Na^+^ excretion in the Na^+^‐depleted group ([K^+^]_norm_/[Na^+^]_low_), but failed to induce detectable natriuresis in the high aldosterone, high KCl group ([KCl]_high_/[Na^+^]_high_) (mean ± SEM, *n* = 6–7 each, Student's paired t‐test, sham: 0.25 mL 0.9% NaCl s.c.). Note the different scaling of the *y*‐axes because of the great differences in salt intake.

Sodium (Na^+^) is the predominant cation of our extracellular space and its homeostasis is tightly regulated by dietary intake in form of salt on the one hand and by the excretion of Na^+^ and chloride (Cl^−^) in the kidney on the other hand. Balanced Na^+^ levels are imperative for the maintenance of the extracellular volume and thereby blood pressure. The steroid hormone aldosterone plays a primary role in maintaining normal body salt homeostasis.^1^ Physiologically, aldosterone synthesis and secretion from the adrenal glands is induced by the hormone angiotensin II (in the setting of decreased extracellular volume) and/or by high levels of plasma K^+^.^2^ In principal cells of the distal nephron, aldosterone couples Na^+^ reabsorption from the urine with K^+^ secretion into the urine (Figure 1A), thereby normalizing both the extracellular volume and plasma K^+^ levels. At the molecular level, these effects are mediated by aldosterone binding to the intracellular mineralocorticoid receptor (MR) in the connecting tubule (CNT) and cortical collecting duct (CCD) of the aldosterone sensitive distal nephron (ASDN).^3^, ^4^ Subsequently, the MR translocates from the cytoplasm to the cell nucleus and induces transcription of the serum and glucocorticoid‐induced kinase (SGK1), the α‐subunit of the amiloride‐sensitive epithelial Na^+^ channel (ENaC) and the Na^+^/K^+^‐ATPase genes.^4^ SGK1 binds to the ubiquitin ligase Nedd4‐2, thereby reducing its interaction with ENaC. This aldosterone‐mediated enhanced expression of ENaC and Na^+^/K^+^‐ATPase results in a strong increase of electrogenic Na^+^ uptake from the urinary tubular lumen via ENaC, thereby increasing the lumen‐negative potential, which is the driving force for both the K^+^ secretion to the urine via renal outer medullary K^+^ channel (ROMK) and for the paracellular Cl^−^ reabsorption into the blood.^5^, ^6^ Therefore, overstimulation of the renal aldosterone signaling pathway, as found in primary aldosteronism, apparent mineralocorticoid excess, or in monogenetic diseases with gain‐of‐function mutations in ENaC (Liddle's syndrome), causes severe salt retention, hypokalemia, and hypertension.^7^

Strikingly, high aldosterone levels due to high dietary K^+^ intake are normally not associated with renal salt retention and disturbances of systemic Na^+^ and Cl^−^ homeostasis, a long known^8^ but until now incompletely understood phenomenon. Actually, K^+^ supplementation even consistently enhances renal Na^+^ excretion in animals as well as in humans, particularly during NaCl loading when Na^+^ delivery to the distal nephron is high.^9^, ^10^, ^11^, ^12^ Mechanistically, recent studies demonstrate that deactivation of the thiazide‐sensitive NaCl cotransporter (NCC) in the distal convoluted tubule (DCT) by an increased plasma [K^+^] contributes to the natriuretic effect of K^+^,^13^, ^14^, ^15^, ^16^, ^17^ but it remains unexplained why this effect is not counterbalanced by an amplified Na^+^ reabsorption via the aldosterone‐MR‐ENaC pathway in the ASDN located downstream.

We therefore questioned whether, besides inactivation of NCC by K^+^ in the DCT, a further mechanism exists, which in the context of high salt and high K^+^ intake counteracts the aldosterone‐MR‐ENaC pathway to prevent subsequent Na^+^ reabsorption in the ASDN.

## 2 METHODS

### 2.1 Ethical approval and experimental animals

All animal protocols were approved by the local authorities (Ministry for Social Affairs, Family, Health and Consumer Protection, Hamburg, Germany, approval number 41/14 and N078/23) and conform to Directive 2010/63/EU of the European Parliament. Experiments were conducted in male C57BL/6 mice (Charles River, Germany) aged 10–16 weeks. Blood withdrawal was performed under deep isoflurane anesthesia (3%–4%). At the end of the experiments, the mice were euthanized by cervical dislocation.

### 2.2 Animal diets

All diets were given for 10 days. The diet of the Na^+^‐depleted group contained 0.93% K^+^ and 0.02% NaCl ([K^+^]_norm_/[Na^+^]_low_) (Ssniff, Germany and Altromin, Germany). The diet of the Na^+^‐replete mice contained 0.93% K^+^ and 3% NaCl ([K^+^]_norm_/[Na^+^]_high_) in the normal K^+^ group and 5% K^+^ and 3% NaCl ([K^+^]_high_/[Na^+^]_high_) in the high K^+^ group (Ssniff, Germany and Altromin, Germany). Increased amounts (5%) of K^+^ were supplied as KCl ([KCl]_high_/[Na^+^]_high_) or as KCitrate ([KCitrate]_high_/[Na^+^]_high_). Mice had free access to water and food and were synchronized to a 12:12 h light–dark cycle. Amounts of food ingested and weight gain of all animals were controlled regularly. During the high K^+^ diets, mice lost weight, but the average weight loss was not statistically different between the different experimental groups and averaged 5.5% in the [KCl]_high_/[Na^+^]_high_ and 4.4% of body weight in the [KCitrate]_high_/[Na^+^]_high_ group.

### 2.3 Antibodies

Antibodies used are described in Table 1.

**TABLE 1:** Antibodies used.

Target	Figure	Source	ID	Application	Dilution
MR	2, 4, 7	Celso Gomez‐Sanchez Lab		IF	1:25
Aquaporin‐2	2, 3, 4, 5, 6	Santa Cruz	SC 9882	IF	1:400
α‐ENaC	3	Jan Loffing Lab		IF	1:100
γ‐ENaC	5	Stressmarq Bioscience Inc.	SPC 405D	IF	1:100
ROMK	6	Jan Loffing Lab		IF	1:400
α‐ENaC	3, 5	Jan Loffing Lab		WB	1:3000
β‐ENaC	3, 5	Stressmarq Bioscience Inc.	SPC 404D	WB	1:20000
γ‐ENaC	3, 5	Stressmarq Bioscience Inc.	SPC 405D	WB	1:5000
ROMK	6	Jan Loffing Lab		WB	1:3000
GAPDH	3, 5, 6	Santa Cruz	SC 365062	WB	1:3000

### 2.4 Renin activity, aldosterone, blood gas analyses, and plasma and urine electrolytes after diet

At day 10 of diet, blood was collected from the retro‐orbital plexus under deep isoflurane anesthesia 1 h before the light was turned off. For determining plasma renin activity, the mice were preconditioned to anesthesia handling. Urine was collected by puncture of the bladder. Afterward, mice were euthanized, the kidneys were removed, and tissue samples from the renal cortex were immediately frozen in liquid N_2_ for further protein isolation, or kidneys were perfusion‐fixed with 4% paraformaldehyde and embedded in paraffin for immunofluorescence analyses. Blood gas analysis and determination of plasma electrolyte concentrations were performed with an ABL 90 series blood gas analyzer (Radiometer, Denmark). Plasma renin activity was assessed as formation of Angiotensin I (Ang I).^18^ The generated Ang I (ng/mL/h, Phoenix Pharmaceuticals Inc., USA) and plasma aldosterone levels (DRG Instruments, Germany) were determined by ELISA following the manufacturer's protocol. Urine Na^+^, K^+^, Cl^−^, and creatinine concentrations were determined using Spotchem EL SE‐1520 (Axonlab, Germany) and Reflotron Plus (Roche, Mannheim). The urinary sodium concentration was normalized to the urinary creatinine concentration for each sample.

### 2.5 Urinary Na^+^ excretion and blood gas analysis after acute amiloride treatment

On day 10 after starting the diets, mice received amiloride (Sigma‐Aldrich) at a dose of 1.4 mg/kg body weight s.c. (dissolved in 250 μL 0.9% NaCl) in the first set of experiments (Figure 1). As amiloride did not induce a natriuretic effect in the [KCl]_high_/[Na^+^]_high_ group, in the second set of experiments (Figure 4), mice received amiloride at a higher dose of 3.9 mg/kg body weight s.c. (dissolved in 250 μL 0.9% NaCl) to exclude dose‐dependent effects. Immediately after the injection, mice were housed in metabolic cages with free access to water (Techniplast, Germany) and urine was collected for 4 h. Urinary Na^+^ excretion after sham treatment (250 μL 0.9% NaCl solution s.c.) was evaluated on day 9 after starting the diet.

### 2.6 Cell culture

Mouse cortical collecting duct (mCCDcl1) cells (kindly provided by Edith Hummler, Lausanne, Switzerland) were cultured in growth medium at 5% CO_2_ and 37°C on collagen 1‐coated flasks (Life Technologies, USA). Growth medium was DMEM/F‐12 (Gibco 31331‐028) containing 30 nM dexamethasone (Sigma‐Aldrich), 2% charcoal stripped FCS (Thermo Fisher, USA), 0.87 μM Insulin (Sigma‐Aldrich), 5 μg/mL human apotransferrin (Sigma‐Aldrich), 10 ng/mL EGF (Life Technologies, USA), 1 nM T3 (Sigma‐Aldrich), and 1% penicillin/streptomycin (Life Technologies, USA). For experiments, mCCDcl1 cells were seeded on transwell PE membranes with 0.4 μm pore size (Sigma‐Aldrich, USA) and grown for up to 6 days with medium containing all additives. Two days before the experiments, the medium was switched to DMEM/F‐12 (Gibco 31331‐028 without additives, except 2% FCS) diluted 1:1 with either normal or high Cl^−^‐buffer. One day before the experiments, mCCDcl1 were cultured in DMEM/F‐12 (Gibco 31331‐028 without additives and without FCS) diluted 1:1 with either normal or high Cl^−^‐buffer.^19^ The normal [Cl^−^] buffer contained (in mM) 86 NaCl, 30 NaHCO_3_, 0.9 CaCl_2_, 1 MgCl_2_, 1 Na_2_HPO_4_, 5 KCl, and 30 NaLactate. The high [Cl^−^] buffer contained (in mM) 116 NaCl, 30 NaHCO_3_, 0.9 CaCl_2_, 1 MgCl_2_, 1 Na_2_HPO_4_, and 5 KCl.

To assay the effect of normal or high [Cl^−^] on MR localization, cells were stimulated with 30 nM aldosterone (Sigma‐Aldrich) for 24 h. After aldosterone stimulation, cells were washed with PBS fixed with 4% paraformaldehyde in PBS for 8 min at RT. Following washes in PBS, unspecific binding was blocked in blocking buffer (5% normal horse serum (Vector, USA) in PBS with 0.05% Triton X‐100 (Sigma‐Aldrich)) for 30 min at RT. MR binding was visualized using a biotinylated donkey‐anti mouse secondary antibody (1:400, Jackson ImmunoResearch Laboratories) for 30 min at RT in blocking buffer followed by the TSA amplification kit (NEB, USA) according to the manufacturer's instructions and SA‐FITC 1:200 (Vector, USA). F‐actin was visualized using AF568 Phalloidin (1:200, Molecular probes, USA) and nuclei/DNA were counterstained with DRAQ5 (1:1000, Molecular Probes, USA).

### 2.7 Immunofluorescence

Two micrometer paraffin sections were deparaffinized and antigen retrieval was performed by microwave boiling (10 mM citrate buffer pH 6.1 or 1x Dako target retrieval solution pH 9) for 30 min at 98°C. Unspecific binding was blocked (5% horse serum with 0.05% triton X‐100) for 30 min RT. Primary antibody incubations were performed in blocking buffer overnight at 4°C. AQP‐2 binding was visualized using a Cy3‐anti‐goat secondary antibody 1:400 (Jackson ImmunoResearch Laboratories, Germany) for 30 min at RT. MR binding was visualized using a biotinylated donkey‐anti mouse secondary antibody (1:400, Jackson ImmunoResearch Laboratories) for 30 min at RT in blocking buffer followed by the TSA amplification kit (NEB, USA) according to the manufacturer's instructions and SA‐FITC 1:200 (Vector, USA). α‐ENaC, γ‐ENaC, and ROMK binding were visualized using an AF488 donkey‐anti‐rabbit secondary antibody (1:100 or 1:400, Jackson ImmunoResearch Laboratories) for 30 min at RT. Stainings were analyzed with a confocal microscope LSM meta5 or with an LSM800 with Airyscan 1 (Zeiss, Germany) and LSM980 with Airyscan 2 (Zeiss, Germany) using the LSM and ZenBlue software (Zeiss, Germany). The MR staining intensity was quantified using ImageJ software (NIH, USA). In each mouse kidney, all AQP‐2‐positive cells with a clearly visible nucleus from four or five randomly selected sections were analyzed by investigators blinded with respect to the diet administered.

### 2.8 Immunoblotting

Protein isolation was performed with isolation solution (250 mM sucrose and 10 mM triethanolamine) and freshly added 1% protease and 1% phosphatase inhibitor cocktail (Sigma‐Aldrich, USA). The homogenate was centrifuged at 1000*g* and the supernatant was used for immunoblotting after the determination of protein concentration (Bradford, Bio‐Rad, Germany). Kidney cortex protein (20 μg) was loaded on SDS gels for the determination of α‐, β‐, γ‐ENaC, ROMK, and GAPDH protein abundance. In addition, for γ‐ENaC immunoblots, 20 μg of deglycosylated protein was loaded on the SDS gel. For deglycosylation, samples were first denatured with SDS at 95°C and then incubated with NP‐40 and PGNaseF (Roche) for 2 h at 37°C. After transfer, equal loading of gels was confirmed by reversible Ponceau staining.^20^ Then, blocking and primary antibody incubation was done according to standard protocols. After washing, the membranes were incubated with HRP‐bound secondary antibody. Antibody‐bound HRP was detected by enhanced chemiluminescence (Bio‐Rad, Germany) and documented with an imaging system (Fusion X, Vilbert Lourmat, Germany). Data were analyzed with Bio 1D software (Vilbert Lourmat, Germany). The secondary antibodies were purchased from Thermo Scientific (Waltham, USA) and DakoCytomation (Denmark).

### 2.9 Quantification and statistical analysis

Data were analyzed using GraphPad Prism software (Version 5.0) and shown as means ± SEM. Plasma renin and aldosterone levels were compared using Kruskal–Wallis test followed by Dunn's multiple comparisons test. All other comparisons between three different groups were made with one‐way ANOVA followed by Bonferroni's multiple comparison tests. Two groups were compared with unpaired or paired two‐tailed Student's *t*‐test. *p* < 0.05 was considered statistically significant.

## 3 RESULTS

### 3.1 MR translocation and ENaC activation by aldosterone is repressed in Na^+^‐replete mice treated with a high KCl diet

We first examined whether the aldosterone response to a high K^+^ diet is altered in the Na^+^‐replete state. Mice consuming a high Na^+^ diet were treated with a normal K^+^ diet ([K^+^]_norm_/[Na^+^]_high_) or a high KCl diet ([KCl]_high_/[Na^+^]_high_). The high KCl intake induced a marked increase in circulating aldosterone levels, which was quantitatively similar to that observed in mice subjected to Na^+^ depletion ([K^+^]_norm_/[Na^+^]_low_), a well‐established stimulus of the renin–angiotensin–aldosterone system (Figure 1B). As aldosterone increases ENaC activity in the distal nephron of the kidney, inhibition of ENaC function should result in an amplified natriuretic response under high aldosterone settings (Figure 1C). Administration of the ENaC blocker amiloride (1.4 mg/kg body weight) indeed elicited a fivefold increase in the urinary [Na^+^]/[creatinine] ratio in the Na^+^‐depleted group as expected (Figure 1D). Although creatinine is secreted into the urine by the mouse kidney,^21^ the data support increased natriuresis in the Na^+^‐depleted group. Strikingly, amiloride‐induced natriuresis was not evident in the high KCl group, despite increased aldosterone levels. In fact, the high KCl group exhibited the same response to amiloride as the normal K^+^ group in which aldosterone levels were low (Figure 1D).

**FIGURE 2:**
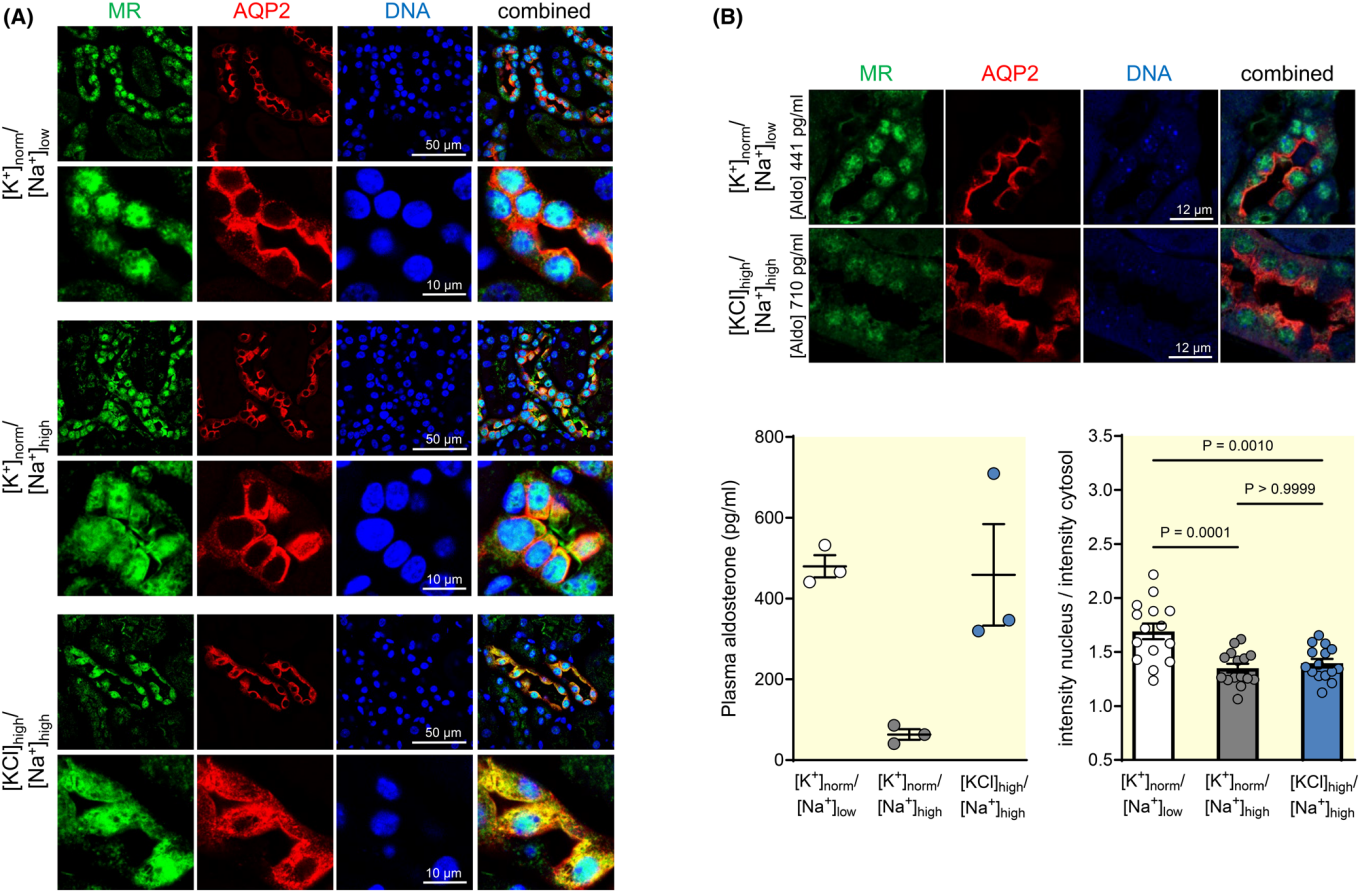
The MR is not translocated to the nucleus in Na^+^‐replete mice receiving a high KCl diet. (A) In the Na^+^‐depleted group ([K^+^]_norm_/[Na^+^]_low_), the mineralocorticoid receptor (MR) immunoreactivity (green) was predominantly detected in the nucleus (blue) of aquaporin‐2 (AQP‐2)‐positive cortical cells (red). In contrast, the high KCl group ([KCl]_high_/[Na^+^]_high_) displayed diffusely dispersed MR immunoreactivity in the cytosol of AQP‐2‐positive cells. (B) In spite of a strong stimulation of aldosterone in the high KCl intake group ([KCl]_high_/[Na^+^]_high_), MR localization to the nucleus is much more prominent in the kidneys of the Na^+^‐depleted group ([K^+^]_norm_/[Na^+^]_low_). Graphs exhibit densitometric quantification of MR staining (ratio of the mean nuclear MR staining intensity to the mean cytosolic MR staining intensity) in mice from the Na^+^‐depleted ([K^+^]_norm_/[Na^+^]_low_) and high KCl ([KCl]_high_/[Na^+^]_high_) groups with matched aldosterone levels (mean ± SEM, *n* = 14 randomly selected sections of each condition, one‐way‐ANOVA).

To elucidate the mechanism underlying the failure of amiloride to induce natriuresis in the high KCl group, we investigated whether aldosterone signaling was changed by assessing the localization of the MR by immunofluorescence in the distal nephron (Figure 2A). After binding of aldosterone, the intracellular MR normally translocates from the cytoplasm to the nucleus where it acts as a transcription factor. Accordingly, the activated MR is predominantly found in the nucleus, whereas nonactivated MR localizes to the cytoplasm. We found an intense MR staining (green) in the nucleus of AQP‐2‐positive cells (red) in the kidney cortex of Na^+^‐depleted mice. As the AQP‐2‐positive cells are the ENaC expressing principal cells, MR translocation in these cells was in agreement with the natriuretic effect of amiloride observed in the Na^+^‐depleted group (Figure 1D). By contrast, in the high KCl group, the MR localization was dispersed over the entire cytoplasm, appearing to be more diffuse within the cell, a distribution similar to that found in the normal K^+^ group (Figure 2A), which had low aldosterone levels (Figure 1B). Quantification of the MR staining in mice with similar aldosterone levels confirmed a significantly increased nuclear intensity in principal cells in the Na^+^‐depleted group but not in the high KCl group, even at very high plasma aldosterone concentration (Figure 2B).

**FIGURE 3:**
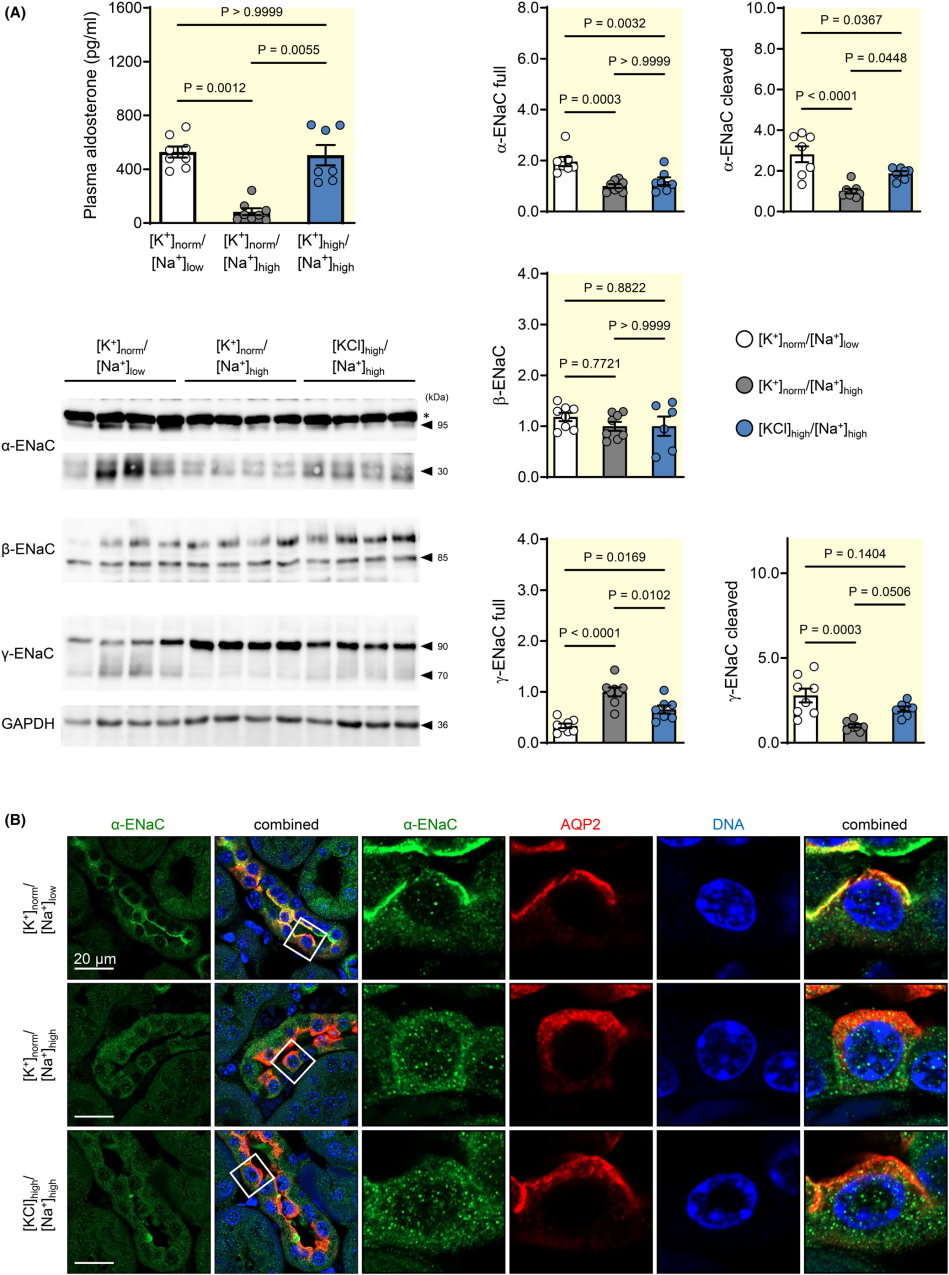
Effect of aldosterone on ENaC protein expression and cellular localization in Na^+^‐replete mice treated with high KCl diet. (A) Protein expression levels of α‐, β‐, and γ‐ENaC subunits in renal cortex after 10 days of indicated diets. In spite of similar plasma aldosterone concentrations, the expression levels of the full (95 kDa) and cleaved (25–30 kDa) forms of α‐ENaC were higher in the Na^+^‐depleted ([K^+^]_norm_/[Na^+^]_low_) than in the high KCl group ([KCl]_high_/[Na^+^]_high_). The proteolytic cleavage form of γ‐ENaC (70 kDa) was unchanged, whereas the full γ‐ENaC (90 kDa) was decreased in the high KCl group ([KCl]_high_/[Na^+^]_high_) as compared with the normal K^+^ group ([K^+^]_norm_/[Na^+^]_high_). Representative immunoblots (with a strong, but unspecific band (*) above 95 kDa in the α‐ENaC immunoblot) and protein levels of α‐, β‐, and γ‐ENaC in kidney cortex are shown. Graphs exhibit densitometric quantification of protein levels (normalized to the levels found in the normal K^+^ group ([K^+^]_norm_/[Na^+^]_high_), mean ± SEM, *n* = 7–8 each diet, one‐way‐ANOVA). (B) The subcellular distribution of the α‐ENaC subunit in mice after 10 days of indicated diets. The α‐ENaC subunit localized in the apical membrane in the Na^+^‐depleted group ([K^+^]_norm_/[Na^+^]_low_), but not in the high KCl group ([KCl]_high_/[Na^+^]_high_) in spite of high aldosterone levels.

Aldosterone primarily enhances ENaC‐dependent Na^+^ transport by increasing amounts of α‐ENaC (95 kDa) protein, presumably due to enhanced transcription, and by raising the abundance of the proteolytically cleaved forms of α‐ENaC (25–30 kDa) and γ‐ENaC (70 kDa) subunits.^22^, ^23^ Stimulation of aldosterone secretion varied markedly between individual mice in response to the high KCl diet (Figure 1B). To avoid a sampling bias, only mice from the high KCl group with a robust aldosterone response to the high KCl diet (comparable to that found in Na^+^‐depleted mice) were further analyzed (Figure 3A). Immunoblots of the three ENaC subunits α, β, and γ in kidney cortex revealed that the protein expression levels of the full and cleaved forms of the α‐ENaC were significantly higher in the Na^+^‐depleted group than in the high KCl group (Figure 3A). The proteolytic cleavage and hence activation of γ‐ENaC (measured as the ratio of the cleaved γ‐ENaC to the full γ‐ENaC isoforms) was also significantly diminished in the KCl group (3.3 ± 0.5, *n* = 7) as compared to the Na^+^‐depleted group (9.7 ± 2.1, *n* = 8).

The observed differences in the functional and cellular response to aldosterone were also reflected by the subcellular localization of α‐ENaC (Figure 3B) in the distal nephron. In AQP‐2‐positive cells, an apical α‐ENaC staining was evident in the Na^+^‐depleted group, whereas in the high KCl group, apical localization of α‐ENaC was mostly absent. Accordingly, in agreement with the missing translocation of the MR to the nucleus a greatly reduced abundance and cleavage of the α‐ and γ‐ENaC subunits and no translocation of α‐ENaC to the apical membrane were observed in the high KCl group.

### 3.2 Lowering the Cl^−^ content of the high K^+^ diet restores MR translocation and ENaC activation by aldosterone in Na^+^‐replete mice

**FIGURE 4:**
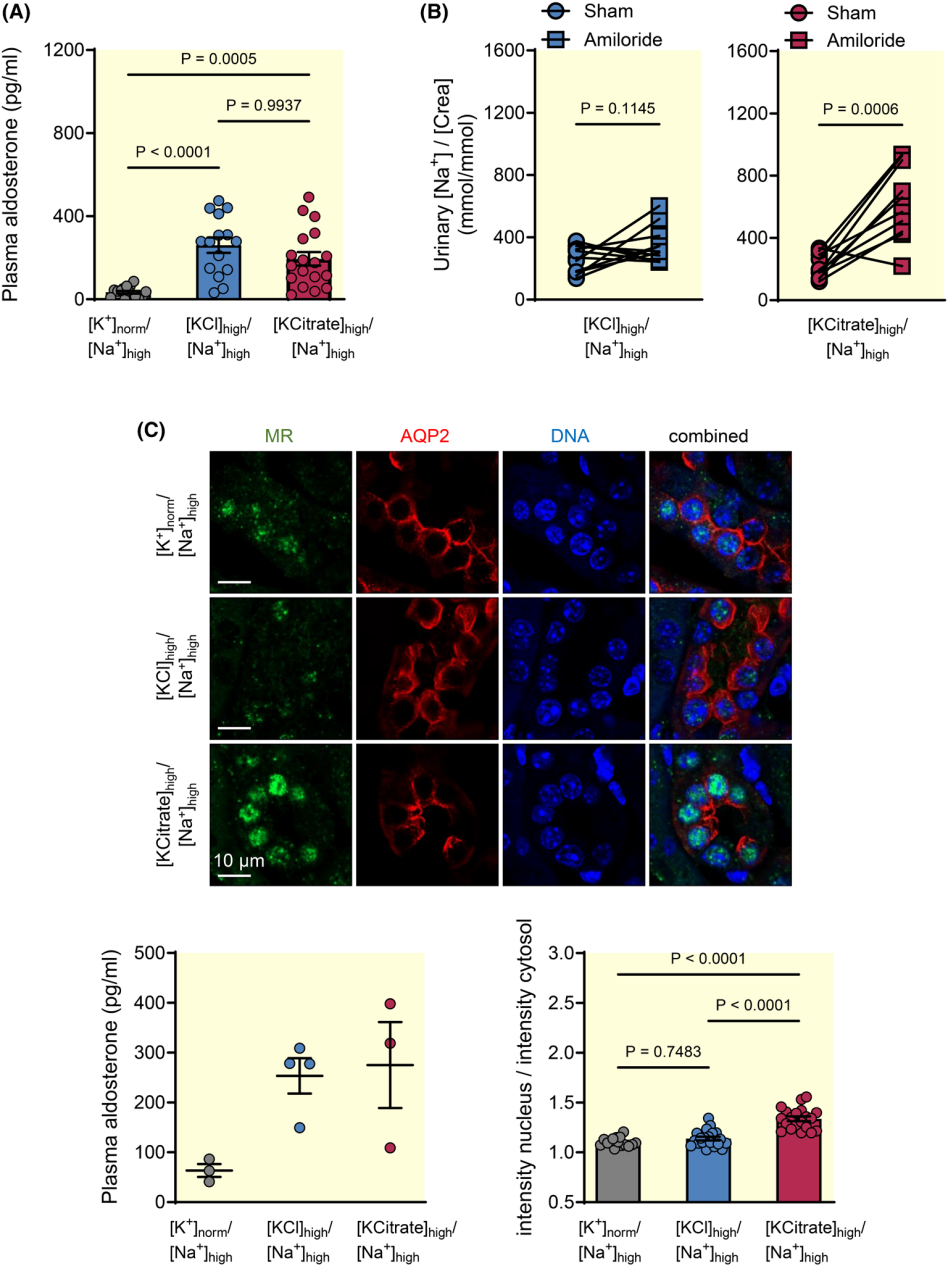
Amiloride induces natriuresis and MR translocation to the nucleus in Na^+^‐replete mice after high KCitrate but not high KCl diet. (A) Plasma aldosterone concentrations in Na^+^‐replete mice fed a normal K^+^ diet ([K^+^]_norm_/[Na^+^]_high_) or two different high K^+^ diets ([KCl]_high_/[Na^+^]_high_ or [KCitrate]_high_/[Na^+^]_high_). Both high K^+^ diet groups exhibited significantly higher plasma aldosterone concentrations than the normal K^+^ diet group (mean ± SEM, *n* = 11–18 each, Kruskal–Wallis test followed by Dunn's multiple comparisons test). (B) Amiloride (3.9 mg/kg s.c.) elicited a significant natriuretic response in the high KCitrate ([KCitrate]_high_/[Na^+^]_high_), but not in the high KCl group ([KCl]_high_/[Na^+^]_high_); mean ± SEM, *n* = 10 or 11, Student's paired *t*‐test). (C) Translocation of the MR (green) to the nucleus (blue) in AQP‐2 positive cortical cells (red) was present in the high KCitrate group ([KCitrate]_high_/[Na^+^]_high_), but not in the high KCl group ([KCl]_high_/[Na^+^]_high_). Plasma aldosterone levels and densitometric quantification of MR staining (ratio of the mean nuclear MR staining intensity to the mean cytosolic MR staining intensity) from 3 to 4 animals of each indicated diet group with matched aldosterone levels (mean ± SEM, *n* = 15–20 randomly selected sections of each condition, one‐way‐ANOVA).

In contrast to the low Na^+^ diet, plasma renin activity and angiotensin II concentrations were not increased in the high KCl group ([KCl]_high_/[Na^+^]_high_). Accordingly, an insufficient stimulation of plasma angiotensin II formation^24^ could account for the observed repression of aldosterone signaling under these conditions. To investigate this possibility, we compared the effects of two high K^+^ diets with varying in Cl^−^ content. To this end, Na^+^‐replete mice received either a high KCl ([KCl]_high_/[Na^+^]_high_) or a high KCitrate ([KCitrate]_high_/[Na^+^]_high_) diet. In both groups, the plasma renin activities were decreased (KCl group: 40.5 ± 8.5 and KCitrate group: 43.1 ± 9.4 AngI ng/mL/h, *n* = 11 each group) and plasma aldosterone concentration increased by similar degrees (Figure 4A).

**FIGURE 5:**
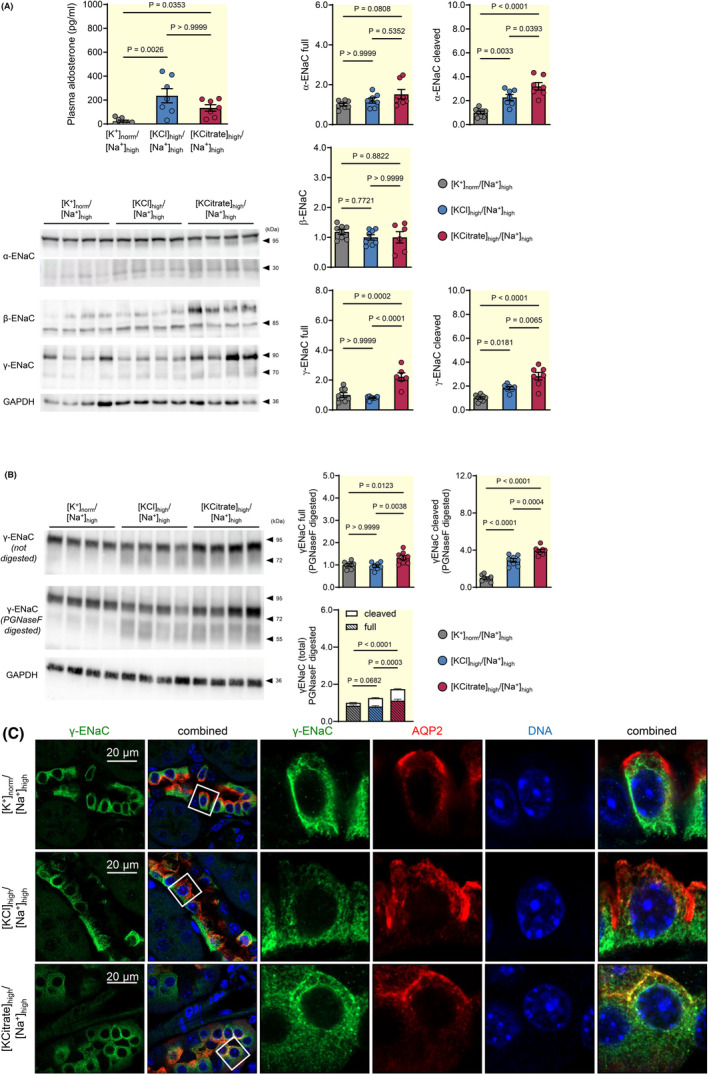
The ENaC protein expression response is restored after high KCitrate diet in Na^+^‐replete mice. (A) Protein expression levels of the cleaved α‐ENaC (25–30 kDa) and γ‐ENaC (70 kDa), and of the full γ‐ENaC (95 kDa) subunits were significantly higher in the high KCitrate group ([KCitrate]_high_/[Na^+^]_high_) compared to the high KCl group ([KCl]_high_/[Na^+^]_high_). Plasma aldosterone concentrations are shown for all mice used for protein expression analysis. Representative immunoblots and renal protein levels of α‐, β‐, and γ‐ENaC in kidney cortex (normalized to the mean level in the normal K^+^ group ([K^+^]_norm_/[Na^+^]_high_)) after 10 days of Na^+^‐replete diet in combination with normal K^+^ ([K^+^]_norm_/[Na^+^]_high_), high KCl ([KCl]_high_/[Na^+^]_high_) or high KCitrate ([KCitrate]_high_/[Na^+^]_high_) diets are shown (mean ± SEM, *n* = 7–8 each diet, one‐way ANOVA). (B) Analysis of γ‐ENaC expression levels after deglycosylation of protein lysates with PNGaseF. Representative immunoblots and renal protein levels of full, cleaved (sum of proximally and distally cleaved), and total (sum of full and cleaved) γ‐ENaC in kidney cortex (normalized to the mean level in the normal K^+^ group ([K^+^]_norm_/[Na^+^]_high_)) after 10 days of indicated diets are shown (mean ± SEM, *n* = 8 each diet, one‐way ANOVA). (C) Subcellular distribution of the γ‐ENaC subunit in the renal cortex of mice in the normal K^+^ ([K^+^]_norm_/[Na^+^]_high_), high KCl ([KCl]_high_/[Na^+^]_high_), and high KCitrate ([KCitrate]_high_/[Na^+^]_high_) group. An apical localization of the γ‐ENaC subunit was observed only in the KCitrate group.

**FIGURE 6:**
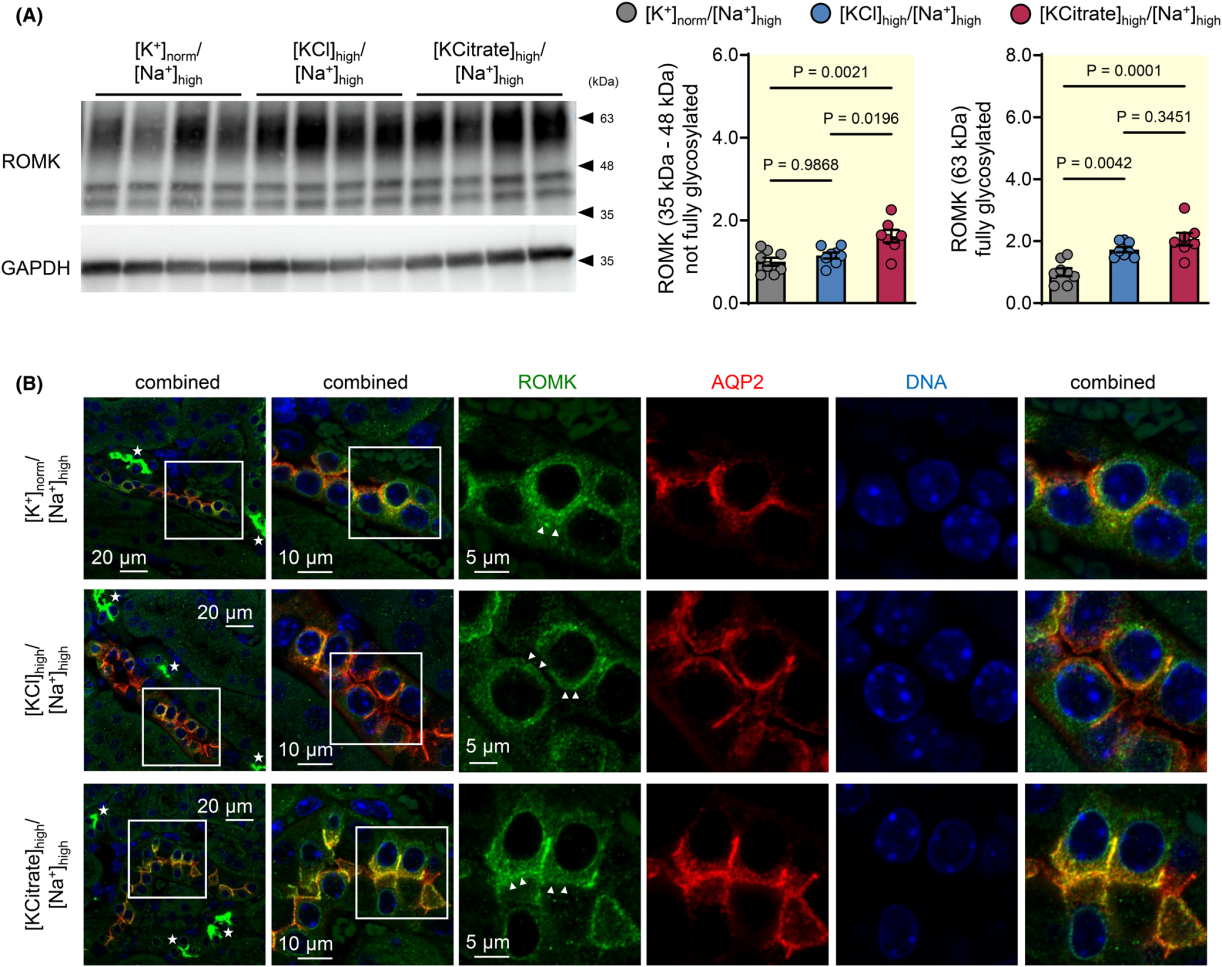
ROMK protein expression is higher after high KCitrate diet than after high KCl diet in Na^+^‐replete mice. (A) Protein expression levels of the un‐glycosylated and core‐glycosylated renal outer medulla K^+^‐channel ROMK (35–48 kDa) were significantly higher in the high KCitrate group ([KCitrate]_high_/[Na^+^]_high_) compared to the high KCl group ([KCl]_high_/[Na^+^]_high_). Representative immunoblots and renal protein levels in kidney cortex (normalized to the mean level in the normal K^+^ group ([K^+^]_norm_/[Na^+^]_high_)) after 10 days of indicated diets are shown (mean ± SEM, *n* = 7–8 each diet, one‐way ANOVA). (B) Subcellular distribution of ROMK in the AQP‐2‐positive cells (red) of the renal cortex of mice in the normal K^+^ ([K^+^]_norm_/[Na^+^]_high_), high KCl ([KCl]_high_/[Na^+^]_high_), and high KCitrate ([KCitrate]_high_/[Na^+^]_high_) group. Apical localization (arrowheads) of ROMK was more evident in the high KCitrate group. It should be noted that the highest levels of ROMK immunofluorescence signals were obtained in AQP‐2‐negative cells (stars).

In contrast to mice of the KCl group, the mice of the KCitrate group displayed the expected functional and cellular response to aldosterone stimulation. At the functional level, a normal response to high aldosterone was demonstrated by a 2.9‐fold increase in urinary [Na^+^]/[creatinine] ratio in response to amiloride administration in the KCitrate group (Figure 4B), demonstrating that the lack of discernible amiloride‐induced natriuresis in the KCl group cannot be explained solely by the high Na^+^ intake and associated high urinary Na^+^ excretion.^25^ At the cellular level, MR translocation to the nucleus was present in AQP‐2‐positive cells of the distal nephron in the KCitrate group (Figure 4C), and protein expression levels of both the full form of γ‐ENaC and the cleaved forms of α‐ and γ‐ENaC were also significantly higher in the KCitrate group than in the KCl group (Figure 5A). Compared to the normal K^+^ group ([K^+^]_norm_/[Na^+^]_high_), a significant increase in the full form of yENaC was observed only in the high KCitrate group. The same observations were made after deglycosylation of the protein samples (Figure 5B). Additionally, γ‐ENaC staining was present at the apical membrane in the KCitrate group, whereas it was mainly detected at the basolateral side of AQP‐2‐positive principal cells in the KCl group (Figure 5B). Protein expression levels (Figure 6A) and apical membrane localization (Figure 6B) of the renal outer medullary K^+^ channel ROMK, which may also be regulated by aldosterone,^26^ were more enhanced in the high KCitrate group than in the high KCl group.

Taken together, these data demonstrate an activation of the aldosterone‐MR pathway in the KCitrate group, whereas in the KCl group, the canonical response to increased aldosterone levels was almost absent.

### 3.3 High extracellular Cl^−^ is sufficient to prevent MR translocation in vitro

The normal response to aldosterone found in the KCitrate group indicates that insufficient stimulation of angiotensin II levels cannot explain the repressed aldosterone response in the KCl group. Since mice in the KCl group ([KCl]_high_/[Na^+^]_high_) received high amounts of Cl^−^, plasma [Cl^−^] was significantly higher in these mice than in mice from the Na^+^‐depleted ([K^+^]_norm_/[Na^+^]_low_) and the high KCitrate group ([KCitrate]_high_/[Na^+^]_high_) (Table 2).

**TABLE 2:** Plasma electrolyte concentrations after 10 days on diets.

Diet group	Mice experimental set 1			Mice experimental set 2		
	Na^+^‐depleted group	Normal K^+^ group	KCl‐group	Normal K+ group	KCl‐group	KCitrate group
	([K^+^]_norm_/[Na^+^]_low_)	([K^+^]_norm_/[Na^+^]_high_)	([KCl]_high_/[Na^+^]_high_)	([K^+^]_norm_/[Na^+^]_high_)	([KCl]_high_/[Na^+^]_high_)	([KCitrate]_high_/[Na^+^]_high_)
Number of animals	15	7	14	6	8	8
Plasma [Na^+^] (mM)	148.4 ± 0.5***	148.7 ± 0.8	153.1 ± 1.0^##^	147.5 ± 0.4	148.5 ± 0.4	149.0 ± 0.7
Plasma [K^+^] (mM)	4.3 ± 0.1	4.3 ± 0.1	4.7 ± 0.2	4.60 ± 0.09	4.57 ± 0.2	4.60 ± 0.1
Plasma [Cl^−^] (mM)	115.3 ± 0.5***	118.7 ± 0.5	121.7 ± 1.6	115.3 ± 0.6	119.6 ± 1.1^##^	110.8 ± 0.6**** ^##^
Plasma [HCO_3_^−^] (mM)	21.2 ± 0.4	20.2 ± 0.6	20.9 ± 0.7	21.1 ± 0.5	19.1 ± 0.8	25.5 ± 0.9**** ^##^
Plasma pH	7.39 ± 0.01	7.36 ± 0.02	7.38 ± 0.01	7.39 ± 0.01	7.35 ± 0.02	7.47 ± 0.01**** ^##^
Hematocrit (%)	46.9 ± 0.4**** ^##^	44.6 ± 0.3	43.7 ± 0.7	44.9 ± 0.4	45.3 ± 0.6	44.6 ± 0.6

*Note*: Significance was calculated by one‐way ANOVA followed by Bonferroni's multiple comparisons test. Significance compared to the high KCl‐group is denoted as ***p < 0.001; ****p < 0.0001. Significance compared to the normal K^+^ is denoted as ^##^ *p* < 0.01.

**FIGURE 7:**
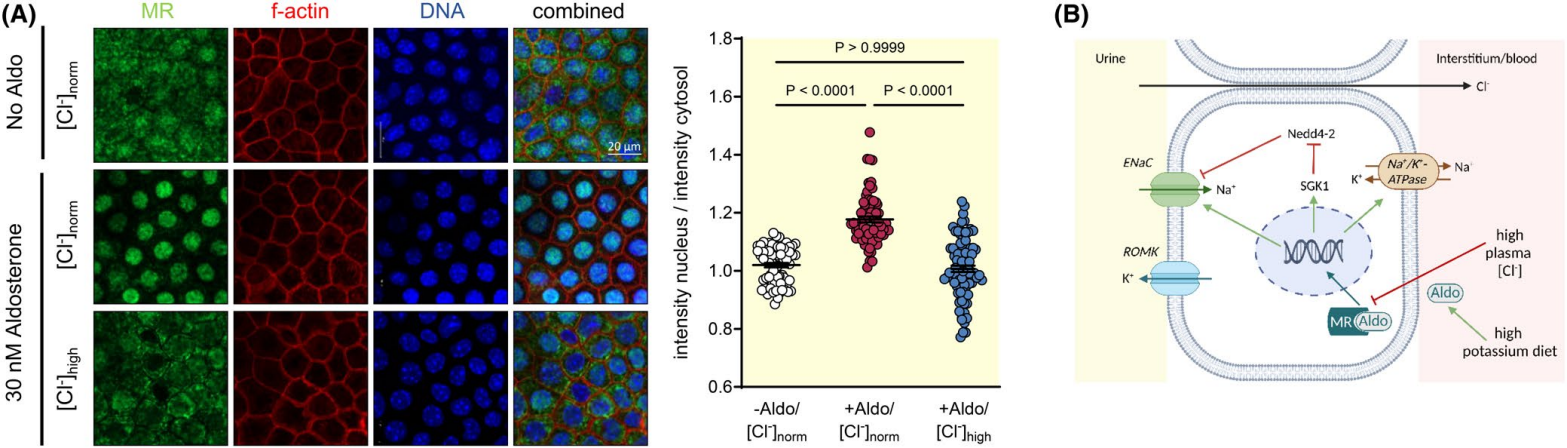
High extracellular [Cl^−^] prevents translocation of the MR to the nucleus in mCCDcl1 cells after aldosterone stimulation. (A) In the absence of aldosterone, the immunoreactivity for mineralocorticoid receptor (MR, green) was diffusely dispersed in mCCDcl1 cells (upper panel). After stimulation with aldosterone (30 nM) for 24 h, MR immunoreactivity was detected in the nucleus (blue) when extracellular [Cl^−^] was normal (115 mM; middle panel). Nuclear MR signal was absent when extracellular [Cl^−^] was high (128 mM; lower panel) despite aldosterone stimulation. The graph shows the densitometric quantification of the MR staining (ratio of the mean nuclear MR staining intensity to the mean cytosolic MR staining intensity, mean ± SEM, *n* = 69–85 cells of each condition, one‐way‐ANOVA). (B) High extracellular chloride inhibits MR translocation to the nucleus despite increased aldosterone levels.

We therefore hypothesized that changes in extracellular [Cl^−^] may directly affect aldosterone signaling in the distal nephron. To test this assumption, we stimulated mCCDcl1 cells,^27^ a cellular model of principal cells in the collecting duct, with aldosterone for 24 h in the presence of normal or high extracellular [Cl^−^]. Since ENaC abundance is sensitive to changes in extracellular [HCO_3_^−^] in the range from 5 to 25 mM,^28^ the [HCO_3_^−^] was clamped to concentrations higher than 25 mM in all experiments (Table 3). At a normal extracellular [Cl^−^] (115 mM), addition of aldosterone (30 nM for 24 h) resulted in an enhanced translocation of MR to the nucleus (Figure 7A, middle panel) as compared to unstimulated cells (Figure 7A, upper panel). MR translocation was completely prevented, when extracellular [Cl^−^] was increased from 115 to 128 mM (Figure 7A, lower panel) using an equimolar design, which allowed to keep [Na^+^], [K^+^], [HCO_3_^−^], pH, and osmolality unchanged (Table 3).

**TABLE 3:** Electrolyte concentrations and pH of normal [Cl^−^] and high [Cl^−^] culture medium.

Culture medium	Normal [Cl^−^]	High [Cl^−^]
[Na^+^] (mM)	151	150
[K^+^] (mM)	5.2	5.4
[Cl^−^] (mM)	115	128
[HCO_3_^−^] (mM)	26.1	25.1
pH	7.44	7.43

*Note*: For alteration of extracellular [Cl^−^], DMEM/F12 medium was diluted with Na‐lactate or NaCl buffer (1:1 medium and buffer). The electrolyte concentrations and pH in the culture media were measured immediately after the experimental period using Spotchem EL SE‐1520 (Axonlab, Germany), ABL 90 series blood‐gas analyzer (Radiometer, Denmark), and standard pH meter (Fa. Mettler Toledo, Germany). [HCO_3_^−^]_c_ was calculated using measured pCO_2_ and pH.

Taken together, the cell culture experiments support the idea that increases of [Cl^−^] in the extracellular milieu are sufficient to impede MR translocation from the cytoplasm to the nucleus in renal principal cells, thereby reducing the stimulatory effect of aldosterone in the distal nephron (Figure 7B).

## 4 DISCUSSION

Our data show a repressive effect of increased extracellular [Cl^−^] on the activation of the aldosterone‐MR‐ENaC pathway in principal cells of the ASDN. Recent studies have demonstrated that lowering extracellular [K^+^] strongly increases Na^+^ reabsorption in the DCT by directly activating the NCC.^14^, ^29^ Together, these findings indicate that salt transport in the distal nephron is highly regulated by the extracellular electrolyte composition. In the DCT, the K^+^ channel Kir4.1/5.1 and the Cl^−^ channel ClC‐K2/b, which localize to the basolateral membrane, translate a fall of extracellular [K^+^] into a reduced intracellular [Cl^−^],^30^ which disinhibits the Cl^−^‐sensitive with no lysine (WNK) kinase 4 leading to NCC activation and Na^+^ retention.^5^, ^17^ In our experiments, repression of the aldosterone‐MR‐ENaC pathway by high [Cl^−^] occurred without alterations of extracellular [K^+^] or [Na^+^] in vitro and in vivo, suggesting the presence of a to date undefined permeability for Cl^−^ in principal cells of the ASDN. Mathematical models of the collecting duct predict that increases of [Cl^−^] in the extracellular space also raise intracellular Cl^−^ levels in principal cells.^31^, ^32^ Therefore, it is conceivable that the inhibitory effect of high extracellular [Cl^−^] could be mediated by the Cl^−^‐sensitive WNK isoforms L‐WNK1 or WNK4 expressed in the ASDN,^33^, ^34^ possibly by affecting aldosterone binding to the MR^35^ or by promoting proteasomal degradation of the MR.^36^, ^37^ Since the distal nephron expresses different isoforms of the Cl^−^/HCO_3_^−^ exchangers^38^ and the lactate receptor GPR81,^39^ changes in intracellular pH secondary to an altered anion exchanger activity may also contribute to the inhibitory effect of high extracellular [Cl^−^] on the aldosterone‐MR‐ENaC pathway.

There is accumulating evidence that the accompanying anion of the K^+^ diet is a critical determinant of transport activity in the distal nephron. A more pronounced upregulation of ENaC and ROMK by K‐alkaline diets in principal cells of the ASDN was also reported recently in mice with a normal Na^+^ intake.^40^, ^41^ The inhibition of the canonical aldosterone‐MR pathway by high [Cl^−^] described here may provide a mechanistic explanation for these differences. Interestingly, even though there was no apparent activation of the aldosterone‐MR pathway in the high KCl group, we observed an upregulation of the cleaved forms of the α‐ENaC and γ‐ENaC. This stimulation of ENaC may be mediated by the aldosterone‐ and MR‐independent mTORC2 pathway, which involves activation of SGK1 and subsequent inhibition of Nedd4‐2.^42^, ^43^ The primary function of this signaling pathway is thought to be the induction of a rapid onset of kaliuresis upon potassium loading,^43^ but it may also play a critical role in the maintenance of K^+^ homeostasis in the absence of MR activation. An increased expression of all three epithelial sodium channel subunits was also recently reported in response to high dietary NaCl intake in hyperaldosteronism with hypokalemia.^25^ The high NaCl intake additionally attenuated the expression of NCC in spite of the severe hypokalemia which would be expected to markedly activate NCC. Together, these observations indicate that the dietary electrolyte composition has a major impact on the regulation of sodium transporters in the distal nephron.

Several studies have documented the beneficial effects of K^+^ supplementation on cardiovascular and renal function, even in the presence of chronic kidney disease (CKD).^44^, ^45^, ^46^ However, K^+^ supplementation also carries a high risk for the development of hyperkalemia in CKD.^47^, ^48^ Since the ability to excrete K^+^ by glomerular filtration or flow‐induced kaliuresis is limited in CKD, urinary K^+^ excretion driven by high ENaC activity assumes a crucial role under these conditions. Accordingly, inhibition of the aldosterone‐MR pathway by high [Cl^−^] would be potentially detrimental for K^+^ homeostasis. The present and other^40^ animal studies suggest that in the clinical setting of CKD, K^+^ supplementation with K‐alkaline diets (KCitrate or KHCO_3_), which are associated with higher activation of ENaC, should be preferred over KCl supplementation.

**FIGURE 8:**
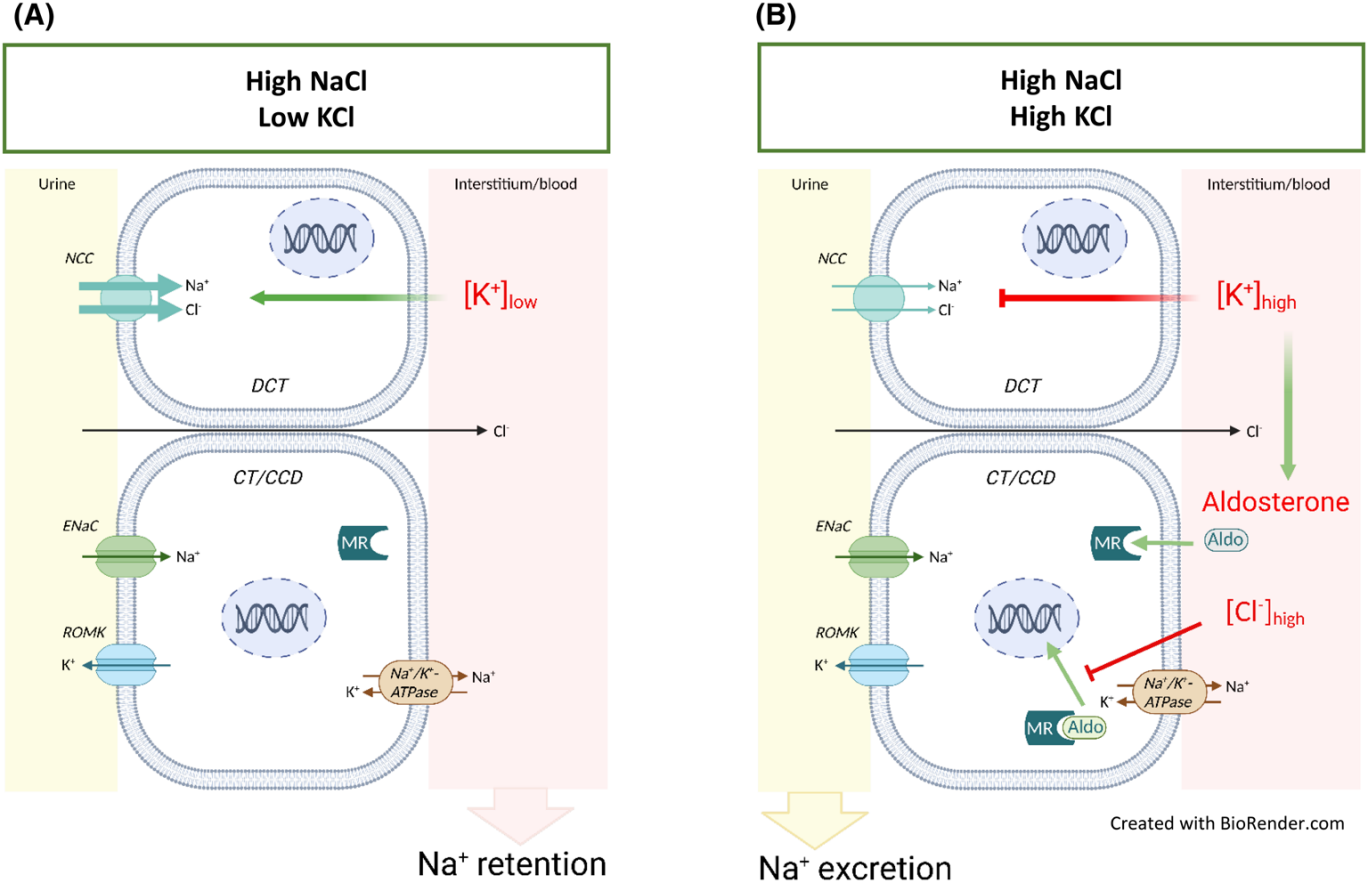
Both inhibition of NCC and silencing of the aldosterone‐MR‐ENaC pathway are required for K^+^‐induced natriuresis. (A) A low K^+^ intake activates NCC in the distal convoluted tubule (DCT) leading to salt retention even when salt intake is high. (B) Increasing K^+^ intake inhibits NCC in DCT, but at the same time stimulates aldosterone release from the adrenal gland. Silencing of the aldosterone‐MR‐ENaC pathway by high extracellular [Cl^−^] prevents Na^+^ reabsorption by ENaC, thus causing salt excretion. Cells of the early distal convoluted tubule (DCT) and principal cells of the connecting tubule (CNT) and of the cortical collecting duct (CCD) are shown. For clarity, both the NCC‐ and ENaC‐expressing cells of the DCT2 and the mTORC2‐dependent stimulation of ENaC^43^ are omitted.

In summary, our findings indicate that the natriuretic effect of increasing dietary K^+^ intake in the Na^+^‐replete state results from a combined inhibition of the K^+^‐regulated NCC and the aldosterone‐regulated ENaC (Figure 8). When NaCl intake is high and K^+^ intake is low, NCC is activated by decreased plasma [K^+^],^14^ while ENaC activity is low in response to suppressed aldosterone levels (Figure 8A). An increased K^+^ intake in the NaCl‐replete state suppresses NCC activity^14^, ^49^ and stimulates aldosterone secretion, but not Na^+^ reabsorption via ENaC because MR signaling in principal cells is attenuated by the high extracellular [Cl^−^] (Figure 8B).

The major physiological function of the extracellular [Cl^−^]‐mediated modulation of the aldosterone‐MR‐ENaC pathway may be to adapt MR activation by a high K^+^ intake to the NaCl balance, thereby preventing excessive MR activation by aldosterone which would result in inappropriate Na^+^ and Cl^−^ reabsorption and its downstream sequel of maladaptive changes in the cardiovascular^50^, ^51^, ^52^ or immune system.^53^ An impairment of the [Cl^−^] sensing mechanism should confer salt sensitivity and may contribute to the deleterious effects of the Western diet on cardiovascular health.

## AUTHOR CONTRIBUTIONS

**Helga Vitzthum:** Conceptualization; investigation; writing – original draft; methodology; validation; visualization; writing – review and editing; formal analysis; project administration; data curation; supervision. **Nina Hauswald:** Investigation; writing – review and editing; methodology; formal analysis. **Helena Pham:** Investigation; methodology; writing – review and editing; formal analysis. **Leya Eckermann‐Reimer:** Investigation; methodology; writing – review and editing. **Catherine Meyer‐Schwesinger:** Investigation; funding acquisition; writing – original draft; methodology; validation; visualization; writing – review and editing; formal analysis; data curation; resources. **Heimo Ehmke:** Conceptualization; writing – original draft; funding acquisition; project administration; supervision; resources; formal analysis.

## FUNDING INFORMATION

This study was funded by the DFG (INST 152/952‐1 FUGG to Catherine Meyer‐Schwesinger) and the DZHK (to Heimo Ehmke).

## CONFLICT OF INTEREST STATEMENT

The authors declare no conflicts of interest.

## Data Availability

The data that support the findings of this study are available on request from the corresponding author.
